# Correction: Krüppel-like factor 3 inhibition by mutated lncRNA *Reg1cp* results in human high bone mass syndrome

**DOI:** 10.1084/jem.2018155403172025C

**Published:** 2025-03-24

**Authors:** Mi Yang, Qi Guo, Hui Peng, Yu-Zhong Xiao, Ye Xiao, Yan Huang, Chang-Jun Li, Tian Su, Yun-Lin Zhang, Min-Xiang Lei, Hui-Ling Chen, Tie-Jian Jiang, Xiang-Hang Luo

Vol. 216, No. 8 | https://doi.org/10.1084/jem.20181554| June 13, 2019

The authors regret that, in their original article, the "OVX+treatment" image provided in Fig. 9 J was mistakenly taken from a different group. This error occurred during figure preparation. The corrected figure is shown here. This correction does not change the original conclusions of the article, and the figure legend remains unchanged. The error appears in print and in PDFs downloaded before March 17, 2025.

**Figure 9.**
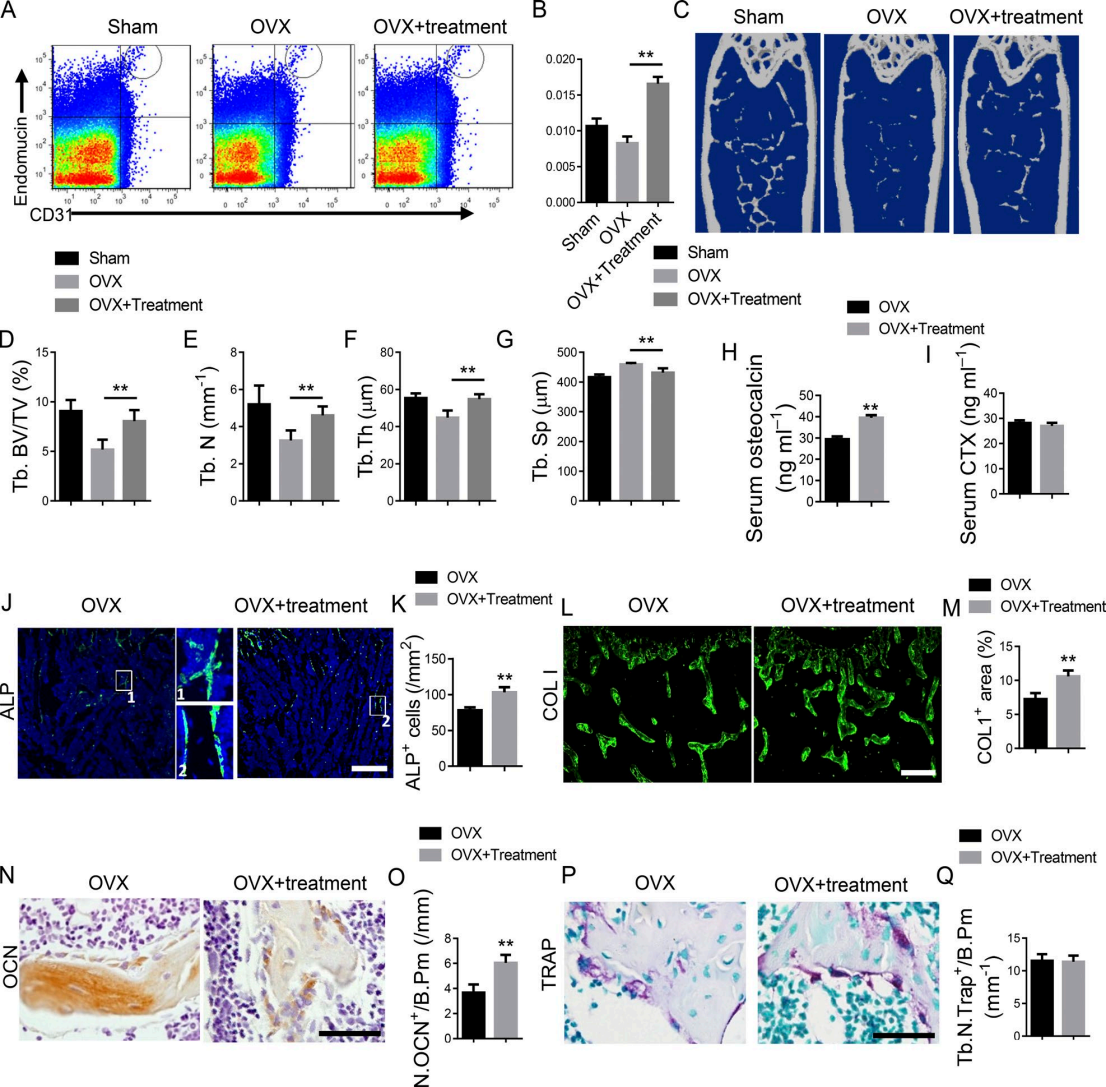
**Ophiopogonin D treatment promotes CD31** ^**hi**^ **EMCN** ^**hi**^ **vessels and bone formation in OVX mice.** 2-mo-old C57/B6 mice underwent OVX surgery and were intraperitoneally treated with Ophiopogonin D at 20 mg/kg every other day for 3 mo. **(A and B)** FACS analysis dot plot (A) and quantification (B) of CD31^hi^EMCN^hi^ ECs (Type H ECs). **(C–G)** Representative μCT images (C) and quantitative μCT analysis (D–G) of trabecular bone microarchitecture in femora. **(H and I)** Serum levels of OCN (H) and CTX (I) at the time of harvest. **(J and K)** Representative images (J) and quantification (K) of ALP (green) immunostaining in femora. Scale bar, 200 µm. **(L and M)** Immunohistochemical staining (L) and quantification (M) of COL 1 (green) in femora. Scale bar, 200 µm. **(N and O)** Immunohistochemical staining (N) and quantification (O) of OCN^+^ cells (brown) in femora. Scale bar, 50 µm. **(P)** Representative images of TRAP staining of femora from Ophiopogonin D–treated mice and their controls. **(Q)** Quantification of TRAP^+^ cells in trabecular bone surfaces. Number of TRAP^+^ cells per bone perimeter (Tb.N.Trap^+^/B.Pm) was measured. Scale bar, 50 µm. (*n* = 6 mice in each group from three independent experiments. Data are shown as the mean ± SD. **, P < 0.01; ANOVA (B and D–G) and Student’s t test (H, I, K, M, O, and Q). Tb. BV/TV, trabecular bone volume per tissue volume; Tb. N, trabecular number; Tb. Sp, trabecular separation; Tb Th, trabecular thickness.

